# New Biomarkers of *Hymenoptera* Venom Allergy in a Group of Inflammation Factors

**DOI:** 10.3390/ijerph18084011

**Published:** 2021-04-11

**Authors:** Kacper Packi, Joanna Matysiak, Eliza Matuszewska, Anna Bręborowicz, Zdzisława Kycler, Jan Matysiak

**Affiliations:** 1 Department of Inorganic and Analytical Chemistry, Poznan University of Medical Sciences, 60-780 Poznan, Poland; kacperpacki1@wp.pl (K.P.); eliza.matuszewska@ump.edu.pl (E.M.); 2 Faculty of Health Sciences, Calisia University, 62-800 Kalisz, Poland; jkamatysiak@gmail.com; 3 Department of Pediatric Pulmonology, Allergy and Clinical Immunology, Poznan University of Medical Sciences, 60-572 Poznan, Poland; abreborowicz@wp.pl (A.B.); zkycler@ump.edu.pl (Z.K.)

**Keywords:** *Hymenoptera* venom allergy, allergic inflammatory response, sting, cytokines, biomarkers

## Abstract

*Hymenoptera* venom allergy significantly affects the quality of life. Due to the divergences in the results of the available test and clinical symptoms of patients, the current widely applied diagnostic methods are often insufficient to classify patients for venom immunotherapy (VIT). Therefore it is still needed to search for new, more precise, and accurate diagnostic methods. Hence, this research aimed to discover new biomarkers of *Hymenoptera* venom allergy in a group of inflammation factors using set of multi-marker Bioplex panel. The adoption of a novel methodology based on Luminex/xMAP enabled simultaneous determination of serum levels of 37 different inflammatory proteins in one experiment. The study involved 21 patients allergic to wasp and/or honey bee venom and 42 healthy participants. According to univariate and multivariate statistics, soluble CD30/tumor necrosis factor receptor superfamily, member 8 (sCD30/TNFRSF8), and the soluble tumor necrosis factor receptor 1 (sTNF-R1) may be considered as effective prognostic factors, their circulating levels were significantly decreased in the allergy group (*p*-value < 0.05; the Area Under the Curve (AUC) ~0.7; Variable Importance in Projection (VIP) scores >1.2). The obtained results shed new light on the allergic inflammatory response and may contribute to modification and improvement of the diagnostic and monitoring methods. Further, large-scale studies are still needed to explain mechanisms of action of studied compounds and to definitively prove their usefulness in clinical practice.

## 1. Introduction

Allergy is a very serious problem over the world. The increasing incidence of all allergic diseases in the last few decades has been confirmed and described in numerous epidemiological studies [1,2]. Next to the food and drug allergy, *Hymenoptera* venom allergic reaction may also be very serious, being one of the most frequent causes of anaphylaxis worldwide [3,4,5,6]. In Central Europe, most post-stinging anaphylactic reactions are caused by honeybees (*Apis mellifera*) and, less frequently, by other *Hymenoptera* such as wasps (*Vespula vulgaris*, or *Vespula germanica*), bumblebees (*Bombus* spp.), or hornets (*Vespa crabro*). An allergic reaction to a sting can occur in people of any age, but it is more common in adults than in children. Men are more exposed to stings than women because they are more likely to work outdoors [7]. According to the current literature, systemic allergic reactions occur in 0.3–7.5% of the population. However, large local reactions concern 2.4–26.4% of people after the sting [7,8,9,10,11]. The group of people particularly exposed to stings due to their work are beekeepers [4,12]. The conducted research suggests that 17–43% of beekeepers are allergic to bee venom. According to the literature, the percentage of systemic reactions in this group ranges from 4.4% to even 43%, while the frequency of severe local reactions is as high as 38% [13,14,15].

The most common mechanism of *Hymenoptera* venom allergy is type I hypersensitivity reaction based on the formation of immunoglobulin E (IgE) class antibodies against the venom. Generally, in the early phase of allergic response, sensitization and the development of memory cells takes place, while the late phase is characterized by the inflammation and tissue injury caused by effector cells action [16]. A key role play T helper 2 (Th2) cells which stimulate B lymphocytes to produce IgE antibodies presented mainly on mast cells [6,16,17]. For an allergic reaction to occur, the allergen must bind to at least two IgE molecules on its surface (known as FcεRI bridging), which initiates the reaction and releases the allergy mediators. There are three groups of these substances: preformed mediators like histamine, heparin, released immediately; mediators produced by the cell after stimulation, such as prostaglandins, thromboxanes, and the third large group of cytokines and chemokines produced and released a little later. Manifestation of the sting may be limited to the local inflammation, but also a wide range of general symptoms could appear, including the immediate life-threatening anaphylactic shock [18,19]. Local reactions can be divided into normal local reactions and large local reactions. Symptoms considered as the correct reaction after a sting include: slight swelling, pain and redness lasting from several minutes to several hours. These symptoms may be more severe if the sting affects the mucosa (e.g., the mouth) or the areas rich in loose connective tissue (eyelids, lips) [4]. Large local reactions are caused by the late phase of the allergic response. They are defined as a swelling that lasts more than 24 h and is more than 10 cm in diameter. The swelling is usually accompanied by local pain, itching, and erythema. Although the reaction is local, it can be life-threatening, particularly if the sting site is in the area of the upper respiratory tract. General symptoms in the course of an allergic reaction after a sting may concern the skin, digestive, respiratory and cardiovascular systems [20]. The skin symptoms include urticaria, pruritus and angioedema, often accompanied by fever. The greatest threat to the patient’s life is laryngeal edema, which may lead to obstruction of the upper respiratory tract or severe bronchospasm. On the part of the gastrointestinal tract, symptoms such as nausea, vomiting, abdominal pain and diarrhea can be observed. The least common, but also the most dangerous, are cardiovascular symptoms. In the course of an allergic reaction to a sting, the following may occur: light-headedness, loss of consciousness, hypotension and, rarely, arrhythmias, and even coronary spasm. Patients often complain of chest pain, which may be also due to the state of great anxiety or panic in the stung person [4]. The most dangerous complication of a systemic allergic reaction is the anaphylactic shock, in the course of which there is a hypotension, loss of consciousness, and finally cardiac, and respiratory arrest. 

The occurrence of severe systemic symptoms in a patient, especially related with the respiratory system, often causes a significant level of fear of the next sting and may significantly affect the quality of life [21]. It is extremely important to properly and immediately diagnose patients to *Hymenoptera* venom allergy and to determine the risk of a future anaphylactic reaction. Nowadays, the base step of diagnostic in *Hymenoptera* venom allergy is a detailed interview. Additional diagnostic tests, like classic skin tests and determination of specific IgE (sIgE) antibodies against the venom in serum, should be performed in individuals who have reported a history of systemic symptoms following a sting [4,22,23]. The diagnostic process of *Hymenoptera* venom allergy usually begins with sIgE measurement against the venom, then prick tests, and in the case of negative results, it is recommended to conduct intradermal tests. It happens (in 15–20% of respondents) that the result of in vitro test is negative, despite the positive medical history and positive skin tests [4,24]. A positive skin test result is obtained in 70–90% of people with a systemic reaction to a sting. If the results of skin tests and the determination of specific IgE antibodies are negative, it is advisable to repeat the above diagnostics after 3–6 months. Studies have shown that the concentration of sIgE is the highest six months after the post-stinging reaction and can then be detected in up to 96% of people allergic to bee venom [25]. The problem with the results of these tests is that they do not correlate with the severity of clinical symptoms [4,26,27]. Serum tryptase determinations are additionally used to assess the risk of severe allergic reactions that may result from another sting [4].

Based on the clinical symptoms, results of diagnostic tests and quality of patient’s life, they are qualified or disqualified for venom immunotherapy (VIT) [21,24]. However, due to the divergences in test results and clinical symptoms of patients, the currently widely applied diagnostic methods are often insufficient to classify patients for this method of treatment [24]. There is extremely important to further develop knowledge in this field and to search for new biomarkers of *Hymenoptera* venom allergy. A promising target in the search for novel biomarkers in allergic diseases have become cytokines and chemokines, which are important mediators of immunity, and their response due to imbalance or deficiency in the cytokine network may largely determine immune disease susceptibility and severity. The whole group of cytokines include interleukins, lymphokines, monokines, interferons (IFN), chemokines, colony stimulating factors (CSF), and a variety of other proteins [26,27]. These proteins play a significant role in the differentiation, maturation, and activation of various immune cells. They may exert pro- and anti-inflammatory effects, depending on the nature of the activating or inhibitory signal and timing [27]. Inflammatory processes caused by the secretion of these cytokines may lead to various manifestations in the skin and other tissues, as well as altered cytokine homeostasis [28]. As the allergic reaction is strictly related to the immune system response, cytokines seem to be a feasible target to investigate as potential biomarkers of the pathophysiological state of allergy too.

This research aimed to discover new biomarkers of *Hymenoptera* venom allergy in a group of inflammation factors, i.e., cytokines. This is the first study which uses set of inflammation multi-marker Bioplex panel for *Hymenoptera* venom allergy examination. Bio-Plex Multiplex immunoassays use fluorescently dyed magnetic beads for the quantification of a biologically relevant target, based on the Luminex/xMAP technology [29]. The adoption of a novel methodology enabled simultaneous determination of serum levels of 37 different inflammation proteins in one experiment. Identifying a specific *Hymenoptera* venom allergy indicator(s) in serum could provide a convenient and non-invasive diagnostic method, help to monitor the treatment or disease progression and possibly enable the development of new treatment strategies.

## 2. Materials and Methods

### 2.1. Study Groups and Serum Samples

The study was approved by the Bioethical Commission of Poznan University of Medical Sciences. The study involved 63 volunteers. Twenty-one of them diagnosed with an allergy to *Hymenoptera* (wasp and honey bee) venom constituted the study group, while 42 healthy participants formed the control group. A total of 30 of all participants were beekeepers: 14 of them suffered from *Hymenoptera* venom allergy, and 16 belonged to the control group. All patients have the main goals of the study and the possible benefits explained. The informed consent was obtained from all volunteers, and in the case of children, from their parents. 

Table 1 shows the demographic profile of all subjects. All participants underwent a detailed medical examination and filled a precise survey. Next, patients were divided into two groups. The division based on venom sIgE levels and clinical symptoms after the sting. Patients from the study group showed allergic reactions after the sting. Symptoms included large local reactions or systemic symptoms with large local reactions. All participants had positive venom specific IgE. The Control group were volunteers who never have been stung by wasp and honeybee in the past, or they had local reactions after the sting (normal reaction or large local reaction). They had negative venom specific IgE diagnostic test—class 0.

**Figure 1:**
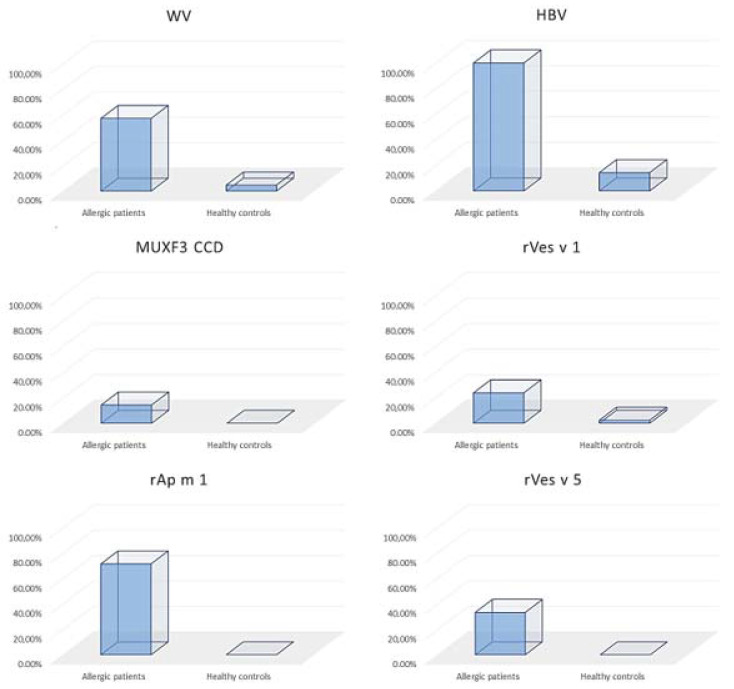
Percentage distribution of positive values of specific IgE (sIgE) within tested groups. sIgE was determined to *WV* wasp venom, *HBV* honeybee venom, *MUXF3 CCD* cross-reactive carbohydrate determinant estimated to MUXF3 from bromelain, *rVes v 1* recombinant phospholipase A1 (*Vespula* spp.), *rAp m 1* recombinant phospholipase A2 (*Apis mellifera*), rVes v 5 recombinant antigen 5 (*Vespula* spp.).

Figure 1 shows patients from study and control groups. In both groups, ImmunoCap (Phadia AB, Uppsala, Sweden) was used to determine sIgE levels. For research purposes were estimated specific IgE to wasp and honeybee venom. Moreover, sIgE to cross-reactive carbohydrate determinant (CCD) were estimated to MUXF3 (neo-glycoprotein fucosylated/xylosylated *N*-glycans) from bromelain. Further sIgE to the species-specific recombinant major allergens (rSSMA) were defined. It includes phospholipase A1 (*Vespula* spp.) (rVes v 1), antigen 5 (*Vespula* spp.) (rVes v 5), and phospholipase A2 (*Apis mellifera*) (rApi m 1). Listed rSSMA were free from CCDs. sIgE values of ≥0.35 kUA/l were considered as positive.

Blood samples obtained from all participants were incubated for 30 min in room temperature for clotting, then centrifuged for 15 min at 4000 rpm. Until analysis, collected sera were stored at −80 °C. 

### 2.2. Measurement of Inflammation Panel

Thirty seven inflammation factors (APRIL/TNFSF13, BAFF/TNFSF13B, sCD30/TNFRSF8, sCD163, Chitinase-3-like 1, gp130/sIL-6Rβ, IFN-α2, IFN-β, IFN-γ, IL-2, sIL-6Rα, IL-8, IL-10, IL-11, IL-12 (p40), IL-12 (p70), IL-19, IL-20, IL-22, IL-26, IL-27 (p28), IL-28A/IFN-λ2, IL-29/IFN-λ1, IL-32, IL-34, IL-35, LIGHT/TNFSF14, MMP-1, MMP-2, MMP-3, Osteocalcin, Osteopontin, Pentraxin-3, sTNF-R1, sTNF-R2, TSLP, TWEAK/TNFSF12) were measured simultaneously using immunoassay Bio-Plex Pro Human Inflammation Panel 1 (Bio-Rad, Hercules, CA, USA) according to the manufacturer’s instructions. The panel of inflammation markers in serum was analyzed using the magnetic separation, flow cytometry method and the Bio-Plex (Bio-Rad) kit: Bio-Plex Pro Human Inflammation Assays. The kit contains reagents, 96-well plate, standards and quality controls recommended for analysis. The technology used in this study is based on fluorescent color-coded magnetic beads connected to specific primary antibodies against proteins, which are listed above. In brief, 50 uL of serum samples, standards and quality controls were added to separate wells containing the antibody-coupled beads and then, the mixture was incubated for 1 h at room temperature. After the incubation period and washing steps, detection antibody-biotin reporters were added to the beads. The establishment of the final reaction mixture was by the addition of fluorescent conjugate streptavidin-phycoerythrin. The concentrations of the inflammation markers were determined based on flow cytometry using Bio-Plex array reader (Bio-Plex MAGPIX, Bio-Rad, Hercules, CA, USA), equipped with two diodes, one of which emits red light with a wavelength of 635 nm, while the other one emits green light with a wavelength of 532 nm. Data acquisition was carried out using Bio-Plex Manager 6.0 software (Bio-Rad, Hercules, CA, USA). Total calibration and verification of the software were performed before the analysis. The standard curve was created from manufacturer-supplied standards. The concentrations of analyzed markers were showed as picograms per milliliter (pg/mL) according to the standard curves. Two from 96 wells contained only Bio-Rad diluents, which were analyzed as blanks. Quality controls (low and high) were used to verify the proper execution of the procedure. 

### 2.3. Data Analysis

To conduct statistical analyses Statistica 13.0 (StatSoft Inc., Tulsa, OK, USA), MedCalc statistical Software (MedCalc Software Ltd, Ostend, Belgium) and MetaboAnalyst 5.0 web platform (www.metaboanalyst.ca) (accessed on 15 January 2021) were used. A value of *p* < 0.05 was considered to be statistically significant. The data were analyzed by applying univariate and multivariate statistical tests. Precise comparison between control and test group were evaluated with t-test or Mann–Whitney test depending on the mode of distribution. The normality of the data distribution was checked using the Shapiro–Wilk test. The Mann–Whitney U test was applied to compare variables without normal distribution and the Levene’s test was used to examine the equality of variances for variables with a normal distribution. If Levene’s test result was not statistically significant (*p* > 0.05), which showed homogeneity of variance between groups, Student’s t-test was conducted. The Welch test was applied if Levene’s test result was statistically significant (*p* < 0.05).

Univariate and multivariate receiver operating characteristic (ROC) curve and Partial-Least Squares Discriminant Analysis (PLS-DA) were calculated by MetaboAnalyst 5.0 web portal and MedCalc Software (MedCalc Software Ltd, Ostend, Belgium). To show the graphical correlation between sensitivity and specificity for each analyte, classical univariate (ROC) curve was evaluated. Moreover, to select and classify the most relevant features, PLS-DA was applied. This method is a standard supervised chemometric analysis that uses a multiple linear regression model to illustrate linear relations between multivariate measurements. The variable’s importance in the PLS-DA model is estimated based on Variable Importance in Projection (VIP) scores. In PLS-DA, the higher the VIP score, the more important is the variable in the classification. It allowed appointing the most relevant inflammation factors in multimarker models. Multivariate ROC was calculated for features with the highest discriminative ability based on partial least squares discriminant analysis algorithm and Monte-Carlo cross-validation. The ROC curves are used to evaluate the sensitivity and specificity of the discriminator. The greater the area under the curve (AUC), the better is the ability of classification of the samples to one of the groups by the model. Before using multivariate statistical analyses, the data were subjected to a process of normalization, transformation, and scaling. 

## 3. Results

### 3.1. Alterations in Serum Concentration of Inflammatory Factors in Hymenoptera Venom Allergy

The applied methodology enabled simultaneous determination of serum levels of multiple inflammation markers in one experiment. The serum concentration of nineteen out of thirty-seven inflammation factors (APRIL/TNFSF13, BAFF/TNFSF13B, sCD30/TNFRSF8, sCD163, Chitinase-3-like 1, gp130/sIL-6Rβ, sIL-6Rα, IL-19, MMP-1, MMP-2, MMP-3, Osteocalcin, Osteopontin, Pentraxin-3, sTNF-R1, sTNF-R2, TSLP, and TWEAK/TNFSF12) were measured successfully (Table 2). The remaining 18 proteins occurred below the lower level of quantification in all analyzed samples or were detected only in part of the samples and therefore, were excluded from further data analysis. Finally, concentrations of the 19 inflammatory factors were subjected to statistical analysis.

The inflammation profile of patients diagnosed with an allergy to *Hymenoptera* (wasp and honey bee) venom was compared to healthy volunteers using Student’s *t*-test, Mann-Whitney or Welch test. The univariate statistics demonstrated that the statistically significant differences between the studied groups occurred in the level of two inflammation markers. In the allergic group, circulating levels of sCD30/TNFRSF8 and sTNF-R1 factors were significantly decreased (*p* < 0.05) in comparison to the control group (Table 3). In the case of other inflammation factors, we also observed differences in their concentrations between the studied groups, although these differences were not statistically significant.

### 3.2. Usefulness of Inflammation Factors in Diagnosis of Hymenoptera Venom Allergy

**Figure 2:**
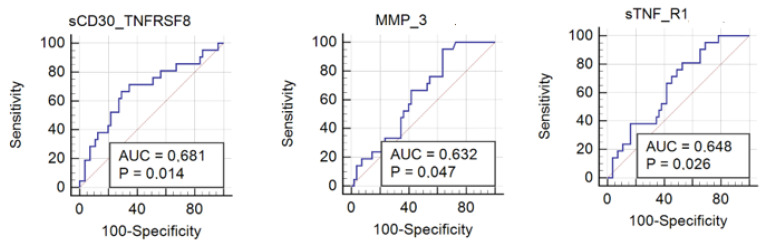
Univariate receiver operating characteristic (ROC) curve representing correlation between serum concentrations of MMP-3, sCD30/TNFRSF8, and sTNF-R1 in allergy patients and control group. (sTNF-R1 sensitivity: 81%, specificity 47.3%; sCD30/TNFRSF8 sensitivity: 66.67%, specificity 70.91%; MMP-3 sensitivity: 95.24%, specificity 36.36%).

**Figure 3:**
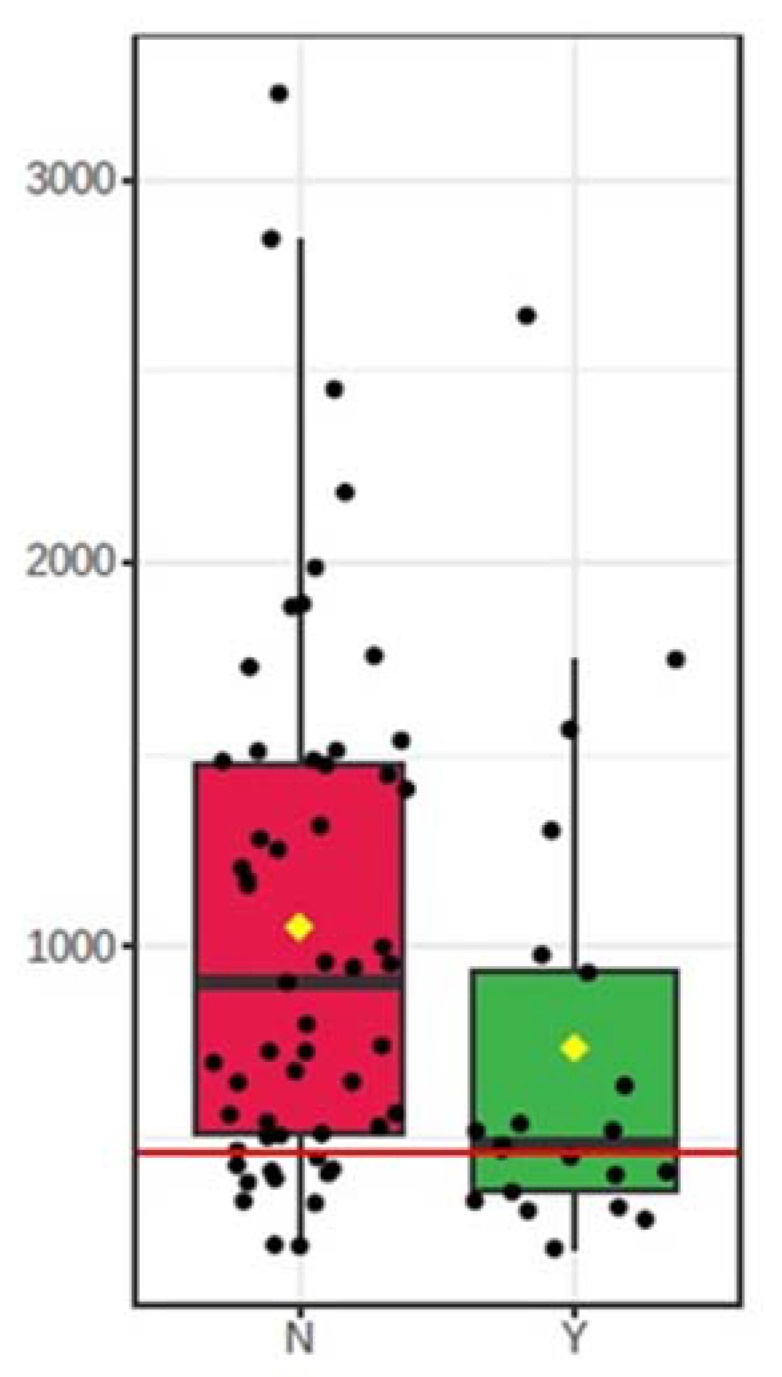
Box-plot of the concentrations of sCD30/TNFRSF8 53 in both groups. A horizontal line is in red, indicating the optimal cut-off. N—control group; Y—allergy group.

**Figure 4:**
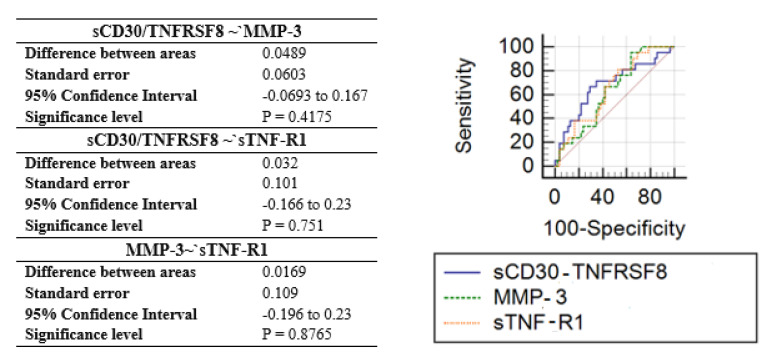
Pairwise comparison of ROC curves.

The discriminative ability of inflammation factors was further checked by calculating the ROC curves. The ROC curve summarizes and gives a graphical presentation of the sensitivity and specificity of the single feature studied factor. Then, accurately classify data can be used to compare the overall accuracy of different biomarkers. In this case, as a satisfactory discriminating factor were considered areas under the curve about 0.7. According to univariate ROC curves, sCD30/TNFRSF8, sTNF-R1, and also MMP-3 protein can be considered as effective prognostic factors of insect venom allergy (Figure 2, Table 4). The highest AUC value (0.681) was obtained by sCD30/TNFRSF8 with the specificity of 70.91% and sensitivity of 66.67% at a cut-off value of 537.62 pg/mL (Figure 3). However, a comparison of ROC curves for all significant proteins showed that MMP-3, sCD30/TNFRSF8, sTNF-R1 are not significantly different from each other as prognostic factors of allergy (Figure 4).

**Figure 5:**
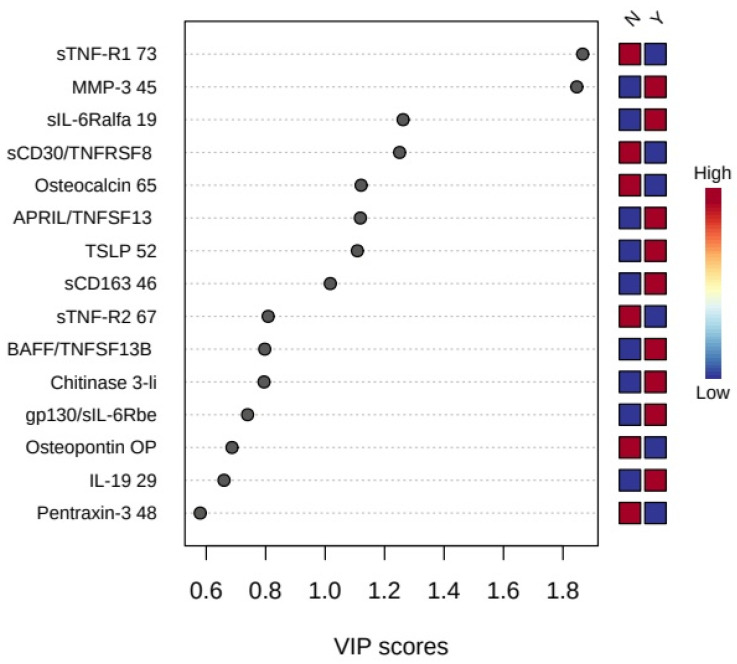
Important features identified by Partial-Least Squares Discriminant Analysis (PLS-DA). The colored boxes on the right indicate the relative concentrations of the corresponding metabolite in each group under study. N—control group; Y—study group.

The data were also analyzed using multivariate statistical analysis. Partial-Least Squares Discriminant Analysis was performed to determine the differentiation ability of inflammation factors profiles and to distinguish the studied groups. The PLS-DA analysis pointed variables, which have the greatest importance in the sample grouping. It has been confirmed that inflammation factors earlier selected in univariate statistics have the best efficacy in discriminating between groups (VIP score > 1.2). Figure 5 shows the variables listed according to their contribution in sample classification. The most differentiating markers were: sTNF-R1, MMP-3, sCD30/TNFRSF8, and sIL-6Ralfa. VIP scores for sTNF-R1 and MMP-3 markers reached a value above 1.8. 

**Figure 6:**
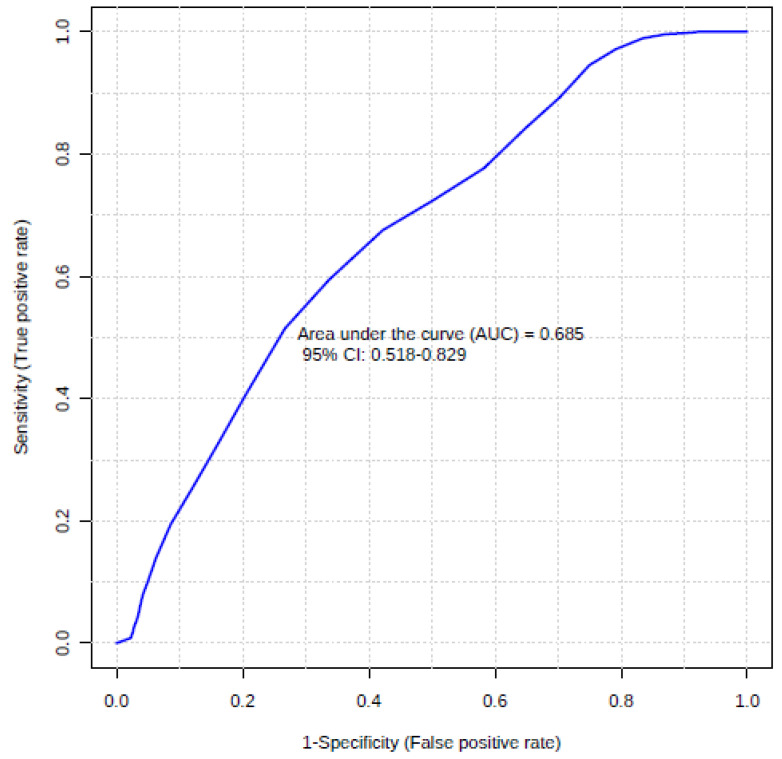
Plot of the multivariate ROC curve for the created biomarker model based on three variables (sTNF-R1, MMP-3, sCD30/TNFRSF8).

In the last step, combinations of three inflammation factors (sTNF-R1, MMP-3, and sCD30/TNFRSF8), earlier selected as significant, were evaluated using multivariate receiver operating characteristic analysis (Figure 6). Model-based on three markers demonstrated slightly higher discriminatory ability (AUC 0.69) than a single marker, sCD30/TNFRSF8 (AUC 0.68). Elimination of any variable or the addition of further variables into the model causes a decrease of the area under the curve. 

## 4. Discussion

Allergies are a very serious problem for many people around the world, and the incidence of these diseases has a tendency to increase in recent years [1,2]. The effects that can be caused by insect stings such as bees and wasps confirm that there is extremely important to further develop knowledge in this field and to search for novel diagnostic methods and markers of *Hymenoptera* venom allergy. The mediators of immunity, like cytokines and chemokines, have become a promising target in the search for new biomarkers in a wide range of allergic diseases. Researchers have shown that many of the cytokines are involved in the inflammatory response associated with insect allergies [16,30,31]. Therefore, our research aimed to discover new biomarkers of *Hymenoptera* venom allergy in a group of inflammation factors. In this study, we successfully evaluated the usefulness of 19 markers in *Hymenoptera* venom allergy diagnostics: APRIL/TNFSF13, BAFF/TNFSF13B, sCD30/TNFRSF8, sCD163, Chitinase-3-like 1, gp130/sIL-6Rβ, sIL-6Rα, IL-19, MMP-1, MMP-2, MMP-3, Osteocalcin, Osteopontin, Pentraxin-3, sTNF-R1, sTNF-R2, TSLP, and TWEAK/TNFSF12. We presented simultaneous measurement of multiple inflammation serum markers based on Bio-Plex technology (Bio-Rad). Unlike ELISA, Bio-Plex allows the rapid (3–4 h) quantitative evaluation of multiple biomarkers using one 96-well plate. In this case, one test allowed for examining the level of 19 proteins at the same time what made this a unique inflammation multi-marker allergy study. The inflammation profile of patients diagnosed with an allergy to *Hymenoptera* (*wasp* and *honey bee*) venom was compared to healthy volunteers. In the allergic group, circulating levels of sCD30/TNFRSF8 and sTNF-R1 were significantly decreased (*p* < 0.05) in comparison to the control group. According to univariate and multivariate ROC curves, which give a graphical presentation of sensitivity and specificity of the studied factors, sCD30/TNFRSF8, sTNF-R1, and MMP-3 protein can be considered as effective prognostic factors of insect venom allergy. 

CD30/TNFRSF8 belongs to the tumor necrosis factor receptor (TNFR) superfamily. sCD30 (soluble CD30) is a soluble form of the CD30 transmembrane protein and is formed by the action of zinc metalloproteinase and is detected in vivo in serum [32,33]. The ligand for this protein is CD30L, otherwise called CD153, which is a membrane-bound cytokine detected on activated lymphocytes, granulocytes, or histiocytes. In physiologic states, the presence of CD30/TNFRSF8 protein was observed on the small subset of activated, not by resting B and T lymphocytes, which is significant for communication between these cell types [34]. Stimulation of CD30 molecules can promote division and survival of T cells, enhance effector activity, including pro-inflammatory cytokine production, and drive the generation of T cell memory [35]. CD30 has different action in various signalling pathways. On the one hand, its stimulation on the cell membrane leads to receptor trimerization and transduction of signal by the recruitment of TNFR. On the other hand, CD30 ligation initiates the mitogen-activated protein kinase (MAPK) pathways, which has a positive effect on the survival of neoplastic cells [34,36]. Increased expression of this protein has been observed in many malignant lymphomas, such as: Hodgkin lymphoma, anaplastic large cell lymphoma (ALCL), gamma-delta-T-cell Lymphoma (GD-TCL), cutaneous lymphomas, or lymphomatoid papulosis (Lyp) [34]. Research suggests that the CD30 antigen can be also recognized as a novel marker of neoplastic mastocytosis in advanced systemic mastocytosis (SM), including aggressive SM (ASM) and mast cell leukemia (MCL) [37,38,39]. Its role as a potential marker has also been suggested in allergic diseases. An elevated level of sCD30 was observed in atopic dermatitis, and it was recognized as a potential marker of clinical severity in this condition. The study proved that sCD30 level was statistically higher in the exacerbation and remission phase of the disease compared to the control group. It was also observed a statistically significant positive correlation between the observed concentration of soluble CD30 receptor and the clinical status of patients in both periods [32,40]. There are also reports that sCD30 level is increased in allergic asthma [41] and that CD30/CD30L interaction is associated with allergic rhinitis [42]. Although there are many scientific reports showing an association between increased expression of this protein and neoplasm or allergic diseases, there is still little research into the expression of CD30 in *Hymenoptera* venom allergy. In one case, it was compared how individual markers on CD4+ T cells changed before and after the venom immunotherapy used to treat patients with *Hymenoptera* venom allergy. Carmen M. Cabrera et al. observed that before VIT there were no significant differences in the level of sCD30 in patients with allergies and in the control group (C = 0.72 ± 0.34 vs. VA = 1.35 ± 0.98), while after VIT immunotherapy, there was a significant decrease in the level of sCD30 compared to results before treatment [17].

TNF-R1, the same as CD30, belongs to TNFR superfamily. The results of our study show, that level of this marker was also significantly decreased in the allergy group when compared to the controls. sTNF-R1 is a soluble form of this protein that is found in blood plasma. TNF-R1 is expressed by most human cells and is one of the two main TNFRs, therefore the functions it performs in the cell are closely related to the functions and activity of TNF in the human body. TNF is recognized as a pro- and anti-inflammatory cytokine, and its expression is promoted mainly by immune system cells [26,43]. The main function of TNF is to stimulate and activate immune cells to the site of infection and to destroy present pathogens like viruses and bacteria. TNF also induces chemokines production, which increases the affinity of leukocyte to their ligands. The research shows that elevated levels of sTNF-R1 are present in many diseases. They are associated with all-cause and cardiovascular mortality in the general population and the occurrence of a heart attack [44,45]. Furthermore, sTNFR-1 was significantly higher in late-stage of bipolar disease (BD). sTNFR1 was also significantly increased in patients with lupus nephritis [46]. On the other hand, there are other studies showing that deletion of TNFR1 causes a lack of contact hypersensitivity in allergic contact dermatitis [47]. Ahmad S. et al. showed that increased expression of TNFR1 also plays a crucial role in allergic inflammatory response through the recruitment of neutrophils, eosinophils, and other lymphocytes [48].

According to the literature, both sCD30 and sTNFR-1 molecules can play a significant role in regulating or activating the allergic inflammatory response [35,41,48,49]. Results of our research show a significant decrease in sCD30 and sTNFR-1 concentration. These observations may be relevant and, in the future, may constitute the basis for modification of the diagnostic process of allergy to *Hymenoptera* venom. sCD30 is considered to be a marker of CD30 expression on Th2 lymphocytes, and thus a marker of Th2 cell activity. Th2 cells play a key role in any allergic reaction, including reaction to *Hymenoptera* venoms [16,50]. CD30 promotes the division and survival of Th2 cells by enhancing their effector activity. The elevated level of CD30 molecules in many allergic and autoimmune diseases suggests an increase in the concentration of this protein also in patients—sufferer from allergy to *Hymenoptera* venom due to increased expression of Th2 cells. However, we received different results, which at the moment remains unclear. It is worth mentioning that earlier studies have shown that the attachment of certain anti-CD30 antibodies induces sCD30 release, whereas other antibodies inhibit this process [51]. Perhaps such a relationship occurs in the case of insect venom allergy, but this phenomenon requires further investigation. Similarly, TNFR-1 as one of the two major receptors for TNF-alpha is a crucial protein in signal transmission from TNF alpha and activation of cellular pathways in many cells involved in the inflammation and allergy process. TNF-alpha activates Th2 cells and its increase was also shown in many allergic diseases. Carballido J.M. et al. reported that venom stimulates Th2 cells to produce small amounts of TNF alpha, which should probably be associated with a slight increase in TNFR1 [52]. However, these results are contrary to ours.

**Figure 7:**
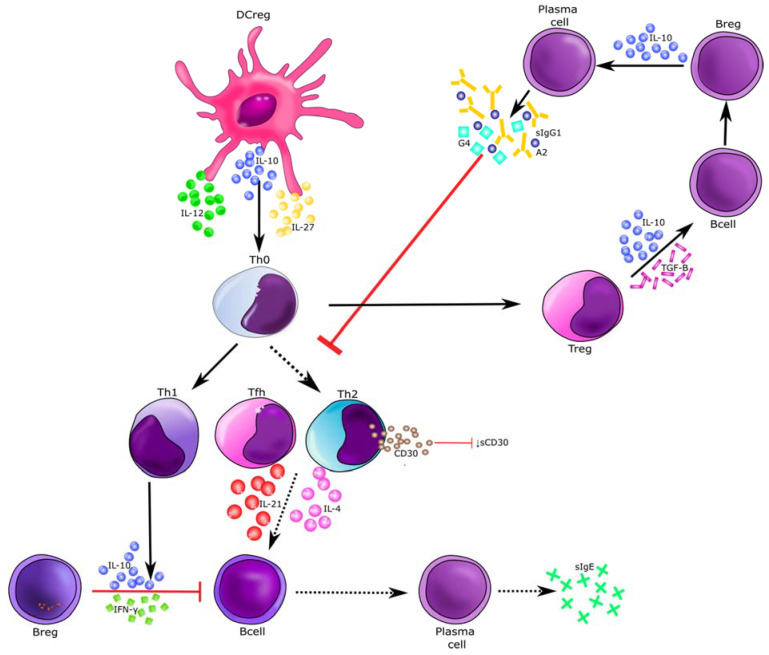
Immunotherapy-induced molecular changes of *Hymenoptera* venom allergy reaction. Red arrows indicate blocking activity induced during venom allergen immunotherapy. *Hymenoptera* venom stimulates dendritic cells and induces Treg, B reg, and other B cells to produce allergen specific IgG1, IgG4, and IgA type bloking antibodies that inhibit the activity of Th2 cells. As a result a shift from Th2 to Th1 type immune deviation occurs. Decrease of Th2 cells level is associated with lowering the expression of CD30 molecules on their surface and consequently leads to reduction of sCD30 in serum [31]. sCD30—soluble CD30; Treg—T regulatory cells; B reg—B regulatory cells; Tfh—T follicular hepler cells; DC reg—regulatory dendritic cells; Th1—T helper 1 cells; Th2—T helper 2 cells.

**Figure 8:**
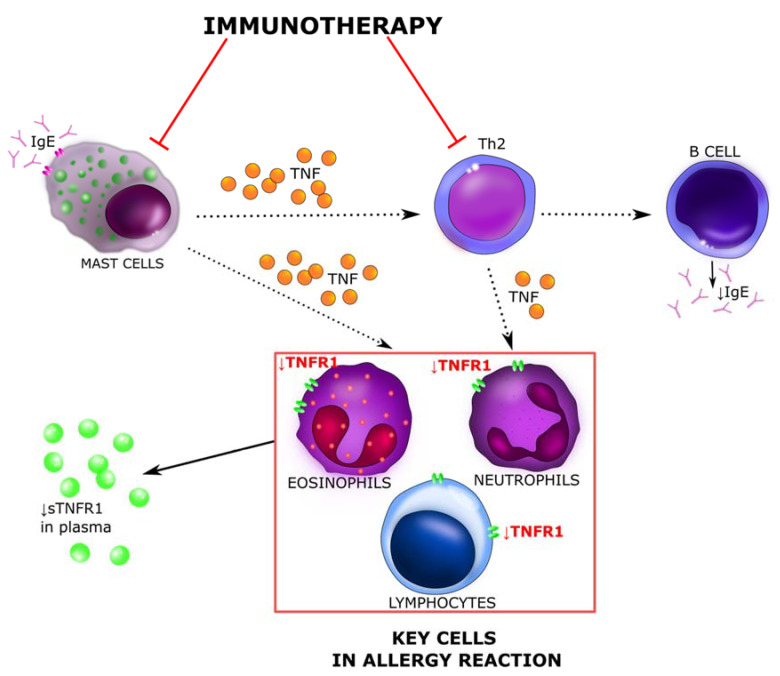
The molecular mechanism determining sTNFR-1 serum levels. Red arrows indicate blocking activity induced during venom allergen immunotherapy. Mast cells play an important role both in IgE-dependent and IgE-independent allergy responses. They have the ability to synthesize and secrete many different cytokines, including IL-3, IL-4, IL-5, and TNF alpha which can extend the allergic reaction. The cytokines and other mediators secreted by mast cells cause an influx of T cells, monocytes and eosinophils into the site of the sting, causing a late-phase reaction (after 6–12 h) dominated by a cellular infiltrate. TNF alpha is a cytokine that plays an important role in allergic reaction. In allergic diseases, elevated levels of TNF reportedly trigger an inflammatory cascade through TNFR1. Immunotherapy causes a decrease in the level and activity of mast cells and Th2 lymphocytes. Both of them secrete TNF, which the major receptor is TNFR1, and is found on most cells of human body. The decrease in TNF production causes a reduction of TNFR1 receptors on the cell membrane and lowers sTNFR1concentration in serum [30,31]. sTNFR-1—soluble tumor necrosis factor receptor 1; Th2—T helper 2 cells.

The decrease in the level of both TNFR1 and sCD30 markers in patients suffering from *Hymenoptera* venom allergy may presumably be associated with taking some medications, i.e., immunosuppressants, tumor necrosis factor alpha blockers or be the result of immunotherapy. In the case of beekeepers allergic to bee venom, prevention and early treatment, i.e., immunotherapy, are crucial. During VIT, induced Treg and B reg cells produce allergen specific IgG1, IgG4, and IgA type blocking antibodies, which in turn inhibit the activity of Th2 cells. VIT causes shift Th2 to Th1 [31]. As sCD30 is a marker of CD30 expression on Th2 lymphocytes, and thus a marker of Th2 cell activity [50], a decrease of Th2 level can cause a reduction of sCD30 in serum, what we observed in this study (Figure 7) [16,17,31]. Research with inhaled allergens has revealed significant growth of blocking antibodies’ serum concentrations, even 100 times in a time and dose dependent manner, due to immunotherapy. While according to the literature, sIgE levels temporarily increases during early phase of subcutaneous VIT and then there is a decrease in serum allergen specific IgE over several year. During immunotherapy, in addition to the decrease in Th2 levels, the number of mast cells also diminishes, and with them there is a reduction in TNF alpha secretion, which may explain the decrease in plasma concentration of sTNFR1 [31]. Based on this, we proposed our own schemes of involvement of the sCD30 and TNF-R1 cytokines in the molecular mechanism of allergy to *Hymenoptera* venom and their actions during the immunotherapy process (Figure 7 and Figure 8).

**Figure 9:**
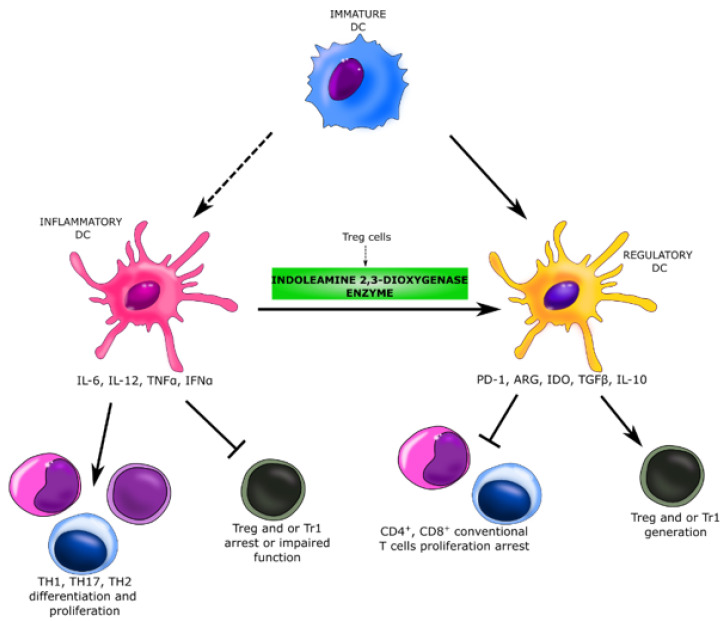
Suppression of the cytokine response in beekeepers. The repeated exposure to bee venom induces Treg cells, which impair the ability of allergen-specific T cells to proliferate and produce Th1 and Th2 cytokines. Treg cells induce indoleamine 2,3-dioxygenase enzyme in dendritic cells what causes the transformation of inflammatory dendritic cells into regulatory dendritic cells. These changes significantly suppress the cytokine response against the major bee venom allergen [16,55]. DC—dendritic cells; Treg—T regulatory cells; Tr1—type 1 regulatory cells; Th17—T helper 17 cells; PD-1—programmed cell death protein 1; ARG—arginase; IDO—indoleamine 2,3-dioxygenase.

Beekeepers are exposed to high levels of bee venom antigens during the beekeeping period. Repeated exposure to the venom allergens reduces T-cell-related cutaneous late-phase reactions and impairs the ability of allergen-specific T cells to proliferate and produce Th1 and Th2 cytokines [16]. In a population of beekeepers shortly after the start of bee venom season, there is observed inhibition of the proliferation of phospholipase A2 (PLA2)-specific effector T cells by Treg cells that produce and secrete IL-10. IL-10 causes the downregulation of MHC-II on antigen-presenting cells (APC), and thus inhibits a wide spectrum of pro-inflammatory cytokines and their receptors. It reduces the production of IL-5 by Th0 and Th2 and downregulates eosinophil activity. Treg cells also influence dendritic cells, they induce an indoleamine 2,3-dioxygenase enzyme, what causes the transformation of inflammatory dendritic cells into regulatory dendritic cells. These changes significantly suppress the antigen-specific proliferative and cytokine responses against PLA2, which is known as the major bee venom allergen. This observations are consistent with our assumptions. Reduction of effector T cells and suppression of the cytokine responses may result in decreased serum concentration of pro-inflammatory cytokines and their receptors, i.e., sCD30 and TNFR1 (Figure 9). This reaction persists as long as bee venom exposure continues. The venom-specific T cell proliferation that is inhibited during exposure period goes back to baseline levels within a few months after the end of the beekeeping season [16,31,53,54].

Moreover, it should be taken into account that most of the previous studies clearly indicate increases in the levels of pro-inflammatory cytokines during an active allergic or autoimmune process, when the immune system is constantly stimulated, and chronic inflammation occurs. The specificity of allergy to *Hymenoptera* venom is different. Patients who are not beekeepers significantly avoid re-contact with the allergen for fear of re-bite and the risk of an anaphylactic reaction [56]. Lack of exposure to an allergenic factor for a long time may cause some variations in the levels of pro-inflammatory cytokines between patients suffering from allergy to *Hymenoptera* venom and to other more frequent and widespread allergens.

This is the first study to determine the level of so many cytokines in the serum of *Hymenoptera* venom allergic patients using the Bio-Plex panel; hence, it is not possible to accurately compare the results with other researchers. Perhaps, the observed differences in the concentrations of pro-inflammatory cytokines involved in the allergic inflammatory response are related to the properties of the insect venom itself. The chemical composition of bee venom is complex, some components possess various and, sometimes, conflicting immune-related effects. Research showed that apamin, histamine, mast cell degranulating peptide, and phospholipase A2 significantly increase the inflammatory response, while other components of bee venom, like the basic polypeptide adolapin, play anti-inflammatory and analgesic actions. Adolapin was also shown to inhibit the activity of PLA2 of bee venom and human lipoxygenase from platelets and possessed antipyretic effects [57]. Moon D.O. et al. reported that treatment of lipopolysaccharide-stimulated BV2 immortalized murine microglial cells with bee venom or the main bee venom peptide melittin, decreased the expression of pro-inflammatory cytokines i.e., IL-1β, IL-6, and TNF-α [58]. The authors linked these anti-inflammatory actions to the leucine zipper sequence in melittin, which includes two leucine residues. It was suggested because Leu-Ala substitution in this sequence gradually diminish this neutralizing effect [57,58].

In this study, we did not observe significant differences in serum level of APRIL/TNFSF13, BAFF/TNFSF13B, sCD163, Chitinase-3-like 1, gp130/sIL-6Rβ, sIL-6Rα, IL-19, MMP-1, MMP-2, Osteocalcin, Osteopontin, Pentraxin-3, sTNF-R2, TSLP, and TWEAK/TNFSF12 protein in patients suffering from *Hymenoptera* venom allergy when compared to healthy volunteers, although these cytokines may be involved in in the course of inflammation general [59,60,61,62,63,64]. Based on univariate statistics, the increased serum level of MMP-3 protein in the study group was also not statistically significant when compared to controls. However, according to univariate and multivariate ROC curves, MMP-3 protein can be considered as effective prognostic factors of insect venom allergy too. Matrix metalloproteinases (MMPs) are a family of zinc-dependent endopeptidases that are related to the degradation of various extracellular components. MMPs are involved in both normal and pathological structural remodeling processes such as tissue repair, cell migration, and tumor necrosis. Noteworthy is the fact that their gene expression is tightly regulated by cytokines that either enhance (IL-1, TNF alpha, and TGF) or inhibit (IL-4) transcription [49]. MMPs are secreted by a number of cell types in allergic diseases such as asthma, including macrophages and leukocytes, and airway structural cells like fibroblasts, epithelial cells, and smooth muscle cells. [65] In healthy tissues, MMP-3 expression is low, but it is increased in tumor formation [66], osteoarthritic changes [67] chronic rhinosinusitis frequently occurring with nasal allergies [68] or severe asthma [65,69]. Dahlen et al. showed that MMP-3 is present in both mast cells and eosinophils, which are key effector of the asthmatic inflammatory response [49]. Furthermore, MMP-3 has been reported in vitro to be involved in the bioprocessing of pro-TNF alpha. Since TNF alpha is localized to human mast cells, this creates the possibility for MMP-3 to act potentially as an activator of TNF alpha release, thereby enhancing the profibrotic course and influencing endothelial cell activation and the recruitment of infiltrating leucocytes in asthma [49,70]. We have not clearly proved the role of MMP-3 protein in the case of allergy to *Hymenoptera* venom. The relationship between MMP-3 and other cytokines and its involvement in the pathomechanism of *Hymenoptera* venom allergy requires further clarification.

The study of the molecular mechanism of the allergy response and the broadening the knowledge on proteins potentially involved in allergic inflammatory is the basis to discover new diagnostics markers that would facilitate but also speed up the currently recommended, sometimes long-term process of confirming patients’ allergies. The results of our research shed a completely new and interesting light on the subject of allergy to *Hymenoptera* venom and the related inflammatory response. In the future sCD30, sTNFR-1 and MMP-3 cytokines may be potential biomarkers in the diagnosis of *Hymenoptera* venom allergy and can help monitor the course of the disease or the effectiveness of treatment. The modification of the diagnostic algorithm of insect venom allergy will accelerate the treatment process of patients and reduce their anxiety and fear related to serious complications caused by the *Hymenoptera* sting. Although, further, more advanced research are needed, possibly on a larger group of patients, which will allow determining the exact molecular pathway in which indicated proteins participate, and thus to find out the cause of different levels of those markers in people with allergy to *Hymenoptera* venom. Understanding the molecular mechanisms of insect venom allergy may be promising in creating new diagnostic methods and qualifying patients for immunotherapy. Additionally, it can contribute to the improvement of their quality of life, reduce the level of anxiety, and even in extreme cases, it will save their lives.

## 5. Conclusions

Expanding the knowledge on allergy to *Hymenoptera* venom is a basis to develop novel, sensitive and specific diagnostic methods. In this study, we successfully investigated the diagnostic usefulness of 19 inflammation markers in patients suffer from *Hymenoptera* venom allergy. The adaptation of a novel immunoassay technique allowed us to assess simultaneously the diagnostic utility of a wide spectrum of compounds involved in the inflammatory response. Our research may contribute to the improvement of *Hymenoptera* venom allergy diagnostic methods and methods of monitoring the course of the disease or effectiveness of treatment. Further and large-scale studies are needed to explain mechanisms of action of studied compounds and to definitively prove their usefulness in clinical practice. In the future, there is also a great need to investigate the serum levels of inflammatory cytokines independently in sensitized beekeepers, patients avoiding re-exposure to the allergenic venom, and patients who have started immunotherapy.

## Figures and Tables

**Table 1:** Characteristics of patients allergic to *Hymenoptera* venom (study group) and healthy individuals (control group).

Characteristics of the Participants	Study Group	Control Group
No. of subjects	21	42
Sex		
Male	12 (57.1%)	26 (61.9%)
Female	9 (42.9%)	16 (38.1%)
Age		
Median	36	54
Mean	37	44
Range	7–67	3–74
Comorbidities		
Diabetes	0	2
Atopic dermatitis	1	0
Allergic rhinitis	3	0
Asthma	2	2
Hypertension	2	5
Atherosclerosis	1	1
Unstable angina	0	1
Medications	9	11
Beekeepers	14 (67%)	16 (38%)
Working period	6 years	28 years
No. of days in the apiary during the beekeeping season/week	2.2	2.7
Stings/day	3	4

**Table 2:** The determined concentrations of 19 inflammation markers in control and studied groups. Concentration values are given in pg/mL.

Inflammation Marker	Allergy Group			Control Group		
	Median	Mean	SD	Median	Mean	SD
APRIL/TNFSF13	26,517.5	25,487.8	16,344.1	17,305.0	24,700.7	21,523.6
BAFF/TNFSF13B	8451.2	8882.3	2076.4	8711.2	9095.3	2857.9
sCD30/TNFRSF8	482.2	735.1	611.7	907.2	1051.6	675.8
sCD163	152,431.4	156,666.3	37,598.5	145,661.2	163,804.5	60,273.9
Chitinase 3-like 1	7308.5	8537.5	3371.8	8727.9	9334.5	4573.4
gp130/sIL-6Rbeta	54,221.1	57,332.8	12,140.3	58,621.7	60,935.2	17,034.5
sIL-6Ralfa	13,706.5	13,199.5	3246.8	12,583.9	12,843.0	4144.3
IL-19	56.7	58.9	9.7	57.9	59.7	15.9
IL-26	59.6	69.0	25.8	70.6	77.1	34.3
MMP-1	1196.9	2091.3	2034.2	1361.2	2097.2	2703.7
MMP-2	24,149.6	26,313.3	12,881.0	26,794.6	30,788.1	20,011.1
MMP-3	5943.1	7032.5	5131.3	3251.1	5248.7	4970.4
Osteocalcin	3997.2	7244.4	7706.6	6967.8	10,220.4	8319.0
Osteopontin	15,388.0	21,273.0	16,831.9	24,705.8	26,392.9	15,668.4
Pentraxin-3	720.0	856.8	459.2	829.1	1069.2	698.0
sTNF-R1	3185.3	3176.3	843.0	3670.8	3860.8	1336.4
sTNF-R2	3904.9	4384.9	1567.3	5167.0	5750.7	3009.7
TSLP	19.7	19.0	4.2	17.3	19.5	6.0
TWEAK/TNFSF12	442.9	485.1	126.8	459.8	514.4	185.1

**Table 3:** Results of univariate statistical analysis of serum inflammation markers in patients with allergy and the control group. *p* values indicates statistical significance.

Inflammation Marker	*p* Value				
	Shapiro–Wilk Test		Mann–Whitney U Test	Student’s *t*-Test	Welch Test
	Allergy Group	Control Group			
APRIL/TNFSF13	0.0970	<0.0001	0.488754		
BAFF/TNFSF13B	0.0033	0.1595	0.890011		
sCD30/TNFRSF8	0.0001	0.0002	0.014876		
sCD163	0.7636	0.0004	0.738166		
Chitinase 3-like 1	0.2398	0.0101	0.644653		
gp130/sIL-6Rbeta	0.1245	0.0260	0.309224		
sIL-6Ralfa	0.6361	0.6943		0.7241	
IL-19	0.1561	0.7654			0.7939 *
IL-26	0.0008	0.0109	0.496046		
MMP-1	0.0001	<0.0001	0.556371		
MMP-2	0.0021	<0.0001	0.556371		
MMP-3	0.0143	0.0001	0.077366		
Osteocalcin	<0.0001	<0.0001	0.083497		
Osteopontin	0.0007	0.0062	0.1379		
Pentraxin-3	0.0022	<0.0001	0.262069		
sTNF-R1	0.4389	0.0108	0.046427		
sTNF-R2	0.2228	0.0033	0.11198		
TSLP	0.0834	0.0001	0.8177		
TWEAK/TNFSF12	0.0055	0.0482	0.556371		

* Welch test assuming unequal variances/Welch test was applied if Levene’s test result was statistically significant (*p* < 0.05).

**Table 4:** Discriminatory value of serum inflammation markers expression showing significant *p*-values (*p* < 0.05) and area under the receiver operating characteristic (ROC) curve (the Area Under the Curve—AUC > 0.630) between studied groups.

Inflammation Marker	Study vs. Control Group	
	*p*-Value	AUC
sCD30/TNFRSF8	0.0135	0.68
sTNF-R1	0.0258	0.65
MMP-3	0.0468	0.63

## Data Availability

The data presented in this study are available in Spreadsheet S1: sIgE levels in study group and control group; Spreadsheet S2: List of concentrations of 37 inflammation markers detected in all samples.

## References

[1] Van Moerbeke D.E. (1997). European allergy white paper: Allergic diseases as a public health problem in Europe. Regional Environmental Change.

[2] I Asher M., Weiland S.K. (1998). The International Study of Asthma and Allergies in Childhood (ISAAC). Clin. Exp. Allergy.

[3] Matuszewska E., Matysiak J., Bręborowicz A., Olejniczak K., Kycler Z., Kokot Z.J., Matysiak J. (2019). Proteomic features characterization of Hymenoptera venom allergy. Allergy Asthma Clin. Immunol..

[4] Matysiak J., Matysiak J., Kokot Z.J., Breborowicz A. (2011). Alergia na jad owadów błonkoskrzydłych ze szczególnym uwzglednieniem pszczoły miodnej (Apis mellifera)—Aktualny stan wiedzy. Alerg. Astma Immunol..

[5] Pesek R.D., Lockey R.F. (2014). Treatment of Hymenoptera venom allergy: An update. Curr. Opin. Allergy Clin. Immunol..

[6] Ollert M., Blank S. (2015). Anaphylaxis to Insect Venom Allergens: Role of Molecular Diagnostics. Curr. Allergy Asthma Rep..

[7] Novembre E., Cianferoni A., Bernardini R., Veltroni M., Ingargiola A., Lombardi E., Vierucci A. (1998). Vierucci Epidemiology of insect venom sensitivity in children and its correlation to clinical and atopic features. Clin. Exp. Allergy.

[8] Grigoreas C., Galatas I.D., Kiamouris C., Papaioannou D. (1997). Insect-venom allergy in Greek adults. Allergy.

[9] Incorvaia C., Mauro M., Pastorello E.A. (1997). Hymenoptera stings in conscripts. Allergy.

[10] Charpin D., Birnbaum J., Lanteaume A., Vervloet D. (1992). Prevalence of allergy to hymenoptera stings in different samples of the general population. J. Allergy Clin. Immunol..

[11] Fernandez J., Blanca M., Soriano V., Sanchez J., Juarez C. (1999). Epidemiological study of the prevalence of allergic reactions to Hymenoptera in a rural population in the Mediterranean area. Clin. Exp. Allergy.

[12] Müller U.R. (2005). Bee venom allergy in beekeepers and their family members. Curr. Opin. Allergy Clin. Immunol..

[13] Münstedt K., Hellner M., Winter D., Von Georgi R. (2008). Allergy to bee venom in beekeepers in Germany. J. Investig. Allergol. Clin. Immunol..

[14] Annila I.T., Karjalainen E.S., A Annila P., A Kuusisto P. (1996). Bee and Wasp Sting Reactions in Current Beekeepers. Ann. Allergy Asthma Immunol..

[15] De la Torre-Morin F., García-Robaina J.C., Vázquez-Moncholí C., Fierro J., Bonnet-Moreno C. (1995). Epidemiology of allergic reactions in beekeepers: A lower prevalence in subjects with more than 5 years exposure. Allergol. Immunopathol..

[16] Fujita H., Soyka M.B., Akdis M., Akdis C.A. (2012). Mechanisms of allergen-specific immunotherapy. Clin. Transl. Allergy.

[17] Cabrera C.M., Urra J.M., Alfaya T., De La Roca F., Feo-Brito F. (2014). Expression of Th1, Th2, lymphocyte trafficking and activation markers on CD4+ T-cells of Hymenoptera allergic subjects and after venom immunotherapy. Mol. Immunol..

[18] Perez-Riverol A., Justo-Jacomini D.L., Zollner R.D.L., Brochetto-Braga M.R. (2015). Facing Hymenoptera Venom Allergy: From Natural to Recombinant Allergens. Toxins.

[19] Sturm G.J., Varga E.-M., Roberts G., Mosbech H., Bilò M.B., Akdis C.A., Antolín-Amérigo D., Cichocka-Jarosz E., Gawlik R., Jakob T. (2018). EAACI guidelines on allergen immunotherapy: Hymenoptera venom allergy. Allergy.

[20] Brown S.G. (2004). Clinical features and severity grading of anaphylaxis. J. Allergy Clin. Immunol..

[21] Antolin-Amerigo D., Ruiz-Leon B., Boni E., Alfaya-Arias T., Alvarez-Mon M., Barbarroja-Escudero J., González-de-Olano D., Moreno-Aguilar C., Rodríguez-Rodríguez M., Sánchez-González M.J. (2018). Component-resolved diagnosis in hymenoptera allergy. Allergol. Immunopathol..

[22] Schiener M., Graessel A., Ollert M., Schmidt-Weber C.B., Blank S. (2017). Allergen-specific immunotherapy of Hymenoptera venom allergy—Also a matter of diagnosis. Hum. Vaccines Immunother..

[23] E Reisman R. (2005). Unusual reactions to insect stings. Curr. Opin. Allergy Clin. Immunol..

[24] Jakob T., Rafei-Shamsabadi D., Spillner E., Müller S. (2017). Diagnostik der Hymenopteren-giftallergie: Aktuelle Konzepte und Entwicklungen mit besonderem Fokus auf die molekulare Allergiediagnostik. Allergy J..

[25] Niedoszytko M., De Monchy J., Van Doormaal J.J., Jassem E., Elberink J.N.G.O. (2009). Mastocytosis and insect venom allergy: Diagnosis, safety and efficacy of venom immunotherapy. Allergy.

[26] Ferreira V.L., Borba H.H., Bonetti A.D.F., Leonart L.P., Pontarolo R. (2019). Cytokines and Interferons: Types and Functions. Autoantibodies and Cytokines.

[27] Zhang J.-M., An J. (2007). Cytokines, Inflammation, and Pain. Int. Anesthesiol. Clin..

[28] Dinarello C.A. (2007). Historical Review of Cytokines. Eur. J. Immunol..

[29] Horala A., Swiatly A., Matysiak J., Banach P., Nowak-Markwitz E., Kokot Z.J. (2017). Diagnostic Value of Serum Angiogenesis Markers in Ovarian Cancer Using Multiplex Immunoassay. Int. J. Mol. Sci..

[30] Voronov E., Apte R.N., Sofer S. (1999). THE systemic inflammatory response syndrome related to the release of cytokines following severe envenomation. J. Venom. Anim. Toxins.

[31] Sahiner U.M., Durham S.R. (2019). Hymenoptera Venom Allergy: How Does Venom Immunotherapy Prevent Anaphylaxis From Bee and Wasp Stings?. Front. Immunol..

[32] Broniarczyk-Dyła G., Prusińska-Bartoś M., Grzegorczyk J., Jarzebska M., Wawrzycka-Kaflik A. (2005). Soluble receptors CD30 and CD26 in serum of patients with atopic dermatitis as markers of disease activity. Adv. Dermatol. Allergol. Dermatol. Alergol..

[33] Hansen H.P., Kisseleva T., Kobarg J., Horn-Lohrens O., Havsteen B., Lemke H. (1995). A zinc metalloproteinase is responsible for the release of cd30 on human tumor cell lines. Int. J. Cancer.

[34] Kampa F., Mitteldorf C. (2021). A review of CD30 expression in cutaneous neoplasms. J. Cutan. Pathol..

[35] Croft M. (2014). The TNF family in T cell differentiation and function--unanswered questions and future directions. Semin. Immunol..

[36] Van der Weyden C.A., Pileri S.A., Feldman A.L., Whisstock J., Prince H.M. (2017). Understanding CD30 biology and therapeutic targeting: A historical perspective providing insight into future directions. Blood Cancer J..

[37] Van Anrooij B., Kluin P.M., Oude Elberink J.N.G., Kluin-Nelemans J.C. (2014). CD30 in systemic mastocytosis. Immunol. Allergy Clin. N. Am..

[38] Chiu A., Orazi A. (2012). Mastocytosis and related disorders. Semin. Diagn. Pathol..

[39] Valent P., Sotlar K., Horny H.-P. (2011). Aberrant expression of CD30 in aggressive systemic mastocytosis and mast cell leukemia: A differential diagnosis to consider in aggressive hematopoietic CD30-positive neoplasms. Leuk. Lymphoma.

[40] Fölster-Holst R., Henseler T., Wehde J., Lemke H., Weichenthal M., Christophers E., Hansen H.P. (2002). Soluble CD30 plasma concentrations correlate with disease activity in patients with atopic dermatitis. Acta Derm. Venereol..

[41] Polte T., Behrendt A., Hansen G. (2006). Direct evidence for a critical role of CD30 in the development of allergic asthma. J. Allergy Clin. Immunol..

[42] Fuchiwaki T., Sun X., Fujimura K., Yamada H., Shibata K., Muta H., Podack E.R., Kawauchi H., Yoshikai Y. (2011). The central role of CD30L/CD30 interactions in allergic rhinitis pathogenesis in mice. Eur. J. Immunol..

[43] Mehta A.K., Gracias D.T., Croft M. (2018). TNF activity and T cells. Cytokine.

[44] Hassan L., Medenwald D., Tiller D., Kluttig A., Ludwig-Kraus B., Kraus F.B., Greiser K.H., Mikolajczyk R. (2020). The association between change of soluble tumor necrosis factor receptor R1 (sTNF-R1) measurements and cardiovascular and all- cause mortality—Results from the population- based (Cardiovascular Disease, Living and Ageing in Halle) CARLA study 2002–2016. PLoS ONE.

[45] Pergola V., Di Salvo G., Martiniello A.R., Irace L., Tedesco M.A., Scialdone A., Iacono A. (2000). TNF alpha and heart failure. Minerva Cardioangiol..

[46] Zheng C., Yan W., Luo Y., Wang T., Shu Y. (2020). Value of sTNF-R1 and linc0597 as indicators for disease activity and diagnosis of lupus nephritis. Eur. Rev. Med. Pharmacol. Sci..

[47] Becke F.M., Hehlgans T., Brockhoff G., Männel D.N. (2001). Development of allergic contact dermatitis requires activation of both tumor necrosis factor-receptors. Eur. Cytokine Netw..

[48] Ahmad S., Azid N.A., Boer J.C., Lim J., Chen X., Plebanski M., Mohamud R. (2018). The Key Role of TNF-TNFR2 Interactions in the Modulation of Allergic Inflammation: A Review. Front. Immunol..

[49] Dahlen B., Shute J., Howarth P. (1999). Immunohistochemical localisation of the matrix metalloproteinases MMP-3 and MMP-9 within the airways in asthma. Thorax.

[50] Pellegrini P., Berghella A.M., Contasta I., Adorno D. (2003). CD30 antigen: Not a physiological marker for TH2 cells but an important costimulator molecule in the regulation of the balance between TH1/TH2 response. Transpl. Immunol..

[51] Horn-Lohrens O., Tiemann M., Lange H., Kobarg J., Hafner M., Hansen H., Sterry W., Parwaresch R.M., Lemke H. (1995). Shedding of the soluble form of CD30 from the Hodgkin-analogous cell line L540 is strongly inhibited by a new CD30-specific antibody (Ki-4). Int. J. Cancer.

[52] Carballido J.M., Carballido-Perrig N., Terres G., Heusser C.H., Blaser K. (1992). Bee venom phospholipase A2-specific T cell clones from human allergic and non-allergic individuals: Cytokine patterns change in response to the antigen concentration. Eur. J. Immunol..

[53] Meiler F., Zumkehr J., Klunker S., Rückert B., Akdis C.A., Akdis M. (2008). In vivo switch to IL-10–secreting T regulatory cells in high dose allergen exposure. J. Exp. Med..

[54] Fallarino F., Grohmann U. (2011). Using an ancient tool for igniting and propagating immune tolerance: IDO as an inducer and amplifier of regulatory T cell functions. Curr. Med. Chem..

[55] Schmidt S.V., Nino-Castro A.C., Schultze J.L. (2012). Regulatory dendritic cells: There is more than just immune activation. Front Immunol..

[56] Oude Elberink J.N.G., Dubois A.E.J. (2003). Quality of life in insect venom allergic patients. Curr. Opin. Allergy Clin. Immunol..

[57] Tusiimire J., Wallace J., Woods N., Dufton M.J., Parkinson J.A., Abbott G., Clements C.J., Young L., Park J.K., Jeon J.W. (2016). Effect of Bee Venom and Its Fractions on the Release of Pro-Inflammatory Cytokines in PMA-Differentiated U937 Cells Co-Stimulated with LPS. Vaccines.

[58] Moon D.-O., Park S.-Y., Lee K.-J., Heo M.-S., Kim K.-C., Kim M.-O., Lee J.-D., Choi Y.H., Kim G.-Y. (2007). Bee venom and melittin reduce proinflammatory mediators in lipopolysaccharide-stimulated BV2 microglia. Int. Immunopharmacol..

[59] Whitehead G.S., Thomas S.Y., Shalaby K.H., Nakano K., Moran T.P., Ward J.M., Flake G.P., Nakano H., Cook D.N. (2017). TNF is required for TLR ligand–mediated but not protease-mediated allergic airway inflammation. J. Clin. Investig..

[60] Huang C.C., Wang C.H., Wu P.W., He J.R., Huang C.C., Chang P.H., Fu C.H., Lee T.J. (2019). Increased nasal matrix metalloproteinase-1 and -9 expression in smokers with chronic rhinosinusitis and asthma. Sci. Rep..

[61] Kim E.G., Na Kim M., Hong J.Y., Lee J.W., Kim S.Y., Kim K.W., Lee C.G., Elias J.A., Song T.W., Sohn M.H. (2020). Chitinase 3-Like 1 Contributes to Food Allergy via M2 Macrophage Polarization. Allergy, Asthma Immunol. Res..

[62] Zhi Y., Gao P., Xin X., Li W., Ji L., Zhang L., Zhang X., Zhang J. (2017). Clinical signifcance of sCD163 and its possible role in asthma (Review). Mol. Med. Rep..

[63] Gowhari-Shabgah A., Shariati-Sarabi Z., Tavakkol-Afshari J., Ghasemi A., Ghoryani M., Mohammadi M. (2020). A significant decrease of BAFF, APRIL, and BAFF receptors following mesenchymal stem cell transplantation in patients with refractory rheumatoid arthritis. Gene.

[64] Konno S., Kurokawa M., Uede T., Nishimura M., Huang S.-K. (2011). Role of osteopontin, a multifunctional protein, in allergy and asthma. Clin. Exp. Allergy.

[65] Ingram J.L., Kraft M. (2015). Metalloproteinases as modulators of allergic asthma: Therapeutic perspectives. Met. Med..

[66] Sage E.H., Reed M., Funk S.E., Truong T., Steadele M., Puolakkainen P., Maurice D.H., Bassuk J.A. (2003). Cleavage of the Matricellular Protein SPARC by Matrix Metalloproteinase 3 Produces Polypeptides That Influence Angiogenesis. J. Biol. Chem..

[67] Stone A.V., Vanderman K.S., Willey J.S., Long D.L., Register T.C., Shively C.A., Stehle J.R., Loeser R.F., Ferguson C.M. (2015). Osteoarthritic changes in vervet monkey knees correlate with meniscus degradation and increased matrix metalloproteinase and cytokine secretion. Osteoarthr. Cartil..

[68] Boruk M., Railwah C., Lora A., Nath S., Wu D., Chow L., Borhanjoo P., Dabo A.J., Chowdhury S., Kaiser R. (2020). Elevated S100A9 expression in chronic rhinosinusitis coincides with elevated MMP production and proliferation in vitro. Sci. Rep..

[69] Hinks T.S., Brown T., Lau L.C., Rupani H., Barber C., Elliott S., Ward J.A., Ono J., Ohta S., Izuhara K. (2016). Multidimensional endotyping in patients with severe asthma reveals inflammatory heterogeneity in matrix metalloproteinases and chitinase 3–like protein 1. J. Allergy Clin. Immunol..

[70] Gearing A.J.H., Beckett P., Christodoulou M., Churchill M., Clements J., Davidson A.H., Drummond A.H., Galloway W.A., Gilbert R., Gordon J.L. (1994). Processing of tumour necrosis factor-α precursor by metalloproteinases. Nat. Cell Biol..

